# Increase in the percentage of obsessive compulsive disorder (OCD) symptoms during the covid pandemic and quarantine at santiago, chile

**DOI:** 10.1192/j.eurpsy.2021.844

**Published:** 2021-08-13

**Authors:** C. Zarate, P. Binder, V. Valdivia, H. Stappung, J.T. Saavedra Perez De Arce

**Affiliations:** 1 School Of Medicine, Department Of Psychiatry, Universidad San Sebastián, Santiago, Chile; 2 Psychiatry, Clínica Psiquiátrica Universitaria, Universidad de Chile, Santiago, Chile

**Keywords:** OCD, COVID, quarentine, Obsessive Compulsive Disorder, lockdown, Chilean experience

## Abstract

**Introduction:**

In pandemic conditions, obsessive rituals such as hygiene can be considered adaptive together with the extreme measures that must be followed to avoid contagion by Covid-19, we suggest that the stress the pandemic has caused may result in an increase in the percentage of OCD symptom and severity in the Chilean population at Santiago.

**Objectives:**

Study OCD symptoms and their severity during a contamination pandemic such as COVID and quarentine, and compare them to national reports of OCD prevalence in Chile. We hypothesize that OCD symptoms would be higher in these stressfull situations.

**Methods:**

An online voluntary and annonymous survey was carried out asking about sociodemographic variables and the Y-BOCKS scale, an OCD symptom severity scale version already validated in Chile.

**Results:**

497 completed the survey and Y-BOCKS scale. 241 people which is equivalent to 48% of the sample presented scores that classified them as having OCD.Off these 30% had mild, 12% moderate and 7% severe symptoms. 85% of them were inquarantine for more than 2 months.

**Figure:**
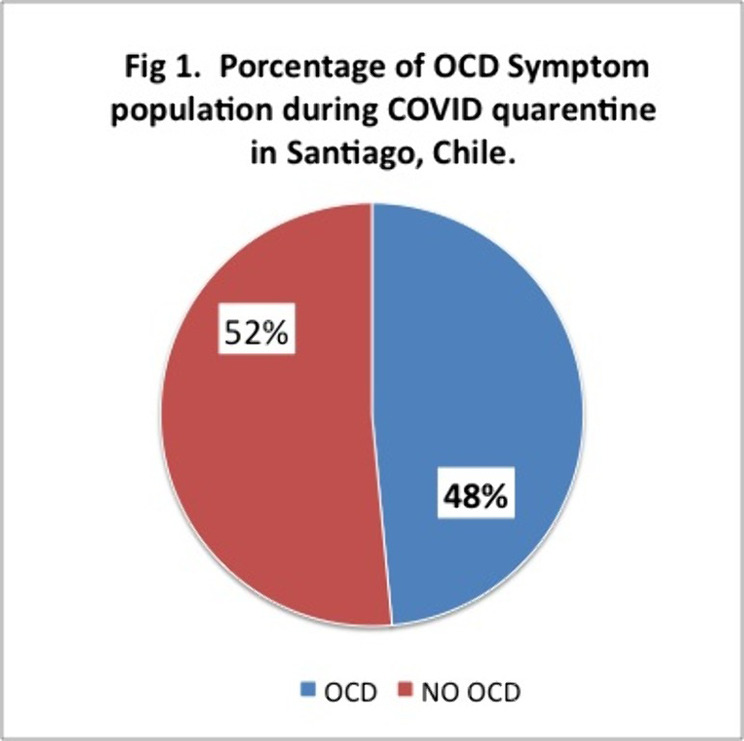

**Figure:**
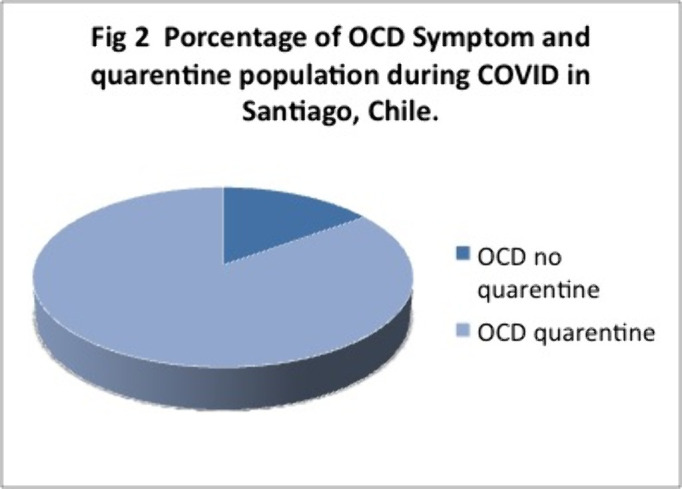

**Conclusions:**

These results are above the 2% of OCD reported at the national level. These percentages may be due to a smaller sample size, but even so, the high percentages of people with symptoms during COVID and those who were in quarentine or lockdown for 2 months or more, stand out. Future analysis and research needs to be made. We ask ourselves wether is Covid, quarentine, or both and of so, how much each pf these contribute to these high percentages of OCD symptoms observed.

